# Beta diversity of aquatic macroinvertebrate assemblages associated with leaf patches in neotropical montane streams

**DOI:** 10.1002/ece3.7215

**Published:** 2021-02-07

**Authors:** Marcos Callisto, Marden S. Linares, Walace P. Kiffer, Robert M. Hughes, Marcelo S. Moretti, Diego R. Macedo, Ricardo Solar

**Affiliations:** ^1^ Laboratório de Ecologia de Bentos Departamento de Genética, Ecologia e Evolução Instituto de Ciências Biológicas Universidade Federal de Minas Gerais Belo Horizonte Brazil; ^2^ Laboratório de Ecologia de Insetos Aquáticos Universidade Vila Velha Vila Velha Brazil; ^3^ Amnis Opes Institute Corvallis OR USA; ^4^ Department of Fisheries & Wildlife Corvallis OR USA; ^5^ Laboratório de Geomorfologia e Recursos Hídricos Departamento de Geografia Instituto de Geociências Universidade Federal de Minas Gerais Belo Horizonte Brazil; ^6^ Departamento de Genética, Ecologia e Evolução Centro de Síntese Ecológica e Conservação Instituto de Ciências Biológicas Universidade Federal de Minas Gerais Belo Horizonte Brazil

**Keywords:** campos rupestres, leaf packs, nestedness, streams, taxa richness, turnover

## Abstract

Over 70% of the total channel length in all river basins is formed by low order streams, many of which originate on mountaintops. Headwater streams play fundamental roles in processing and transporting terrestrial and aquatic organic matter, often harboring high biodiversity in bottom leaf patches deposited from riparian vegetation. The objective of this study was to assess the variation in taxonomic composition (measured by beta diversity of aquatic macroinvertebrates) among stream sites located in the Espinhaço Meridional Mountain Range, part of a UNESCO Biosphere Reserve in eastern Brazil. We tested two hypotheses. (a) Taxa turnover is the main reason for differences in aquatic insect assemblages within stream sites; we predicted that turnover would be higher than nestedness in all stream sites. (b) Stream site altitude and catchment elevation range are the main explanatory variables for the differences in beta diversity; we predicted that local stream site variables would account for only minor amounts of variation. In both dry and wet seasons, we sampled twice in two habitat types (five leaf patches in pools and five in riffles) in each of nine stream sites distributed in three different river basins. We computed average pairwise beta diversity among sampling stations and seasons in each stream site by using Jaccard and Bray–Curtis indices, and calculated the percentages of diversity resulting from turnover and nestedness. Finally, we tested the degree that local‐ or catchment‐level predictor variables explained beta diversity. We found that turnover was the main component of beta diversity and that both dissolved oxygen and elevation range best explained Bray–Curtis beta diversity. These results reinforce the importance of leaf patches in montane (sky islands) Neotropical savanna streams as biodiversity hotbeds for macroinvertebrates, and that both local and landscape variables explained beta diversity.

## Graphical abstract

Headwater streams play fundamental roles in processing and transporting terrestrial and aquatic organic matter, often harboring high biodiversity. Here we assess the variation in taxonomic composition among stream sites located in the Espinhaço Mountain Range, a UNESCO Biosphere Reserve in eastern Brazil. We found that turnover was the main component of beta diversity and that both dissolved oxygen and elevation range explained Bray–Curtis beta diversity.

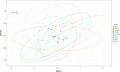

## 1 INTRODUCTION

Facing an accelerated scenario of global warming and considering that mountain streams harbor endemic taxa that may be extirpated as the streams warm (Rivers‐Moore, 2012; Hotaling et al., 2017), mountaintop ecosystems are critical for two reasons. They support a unique and specialized biodiversity (Colvin et al., 2019) and offer refuges for aquatic species that could potentially migrate from lower reaches with unsuitably warm water, to upper reaches with cooler conditions (Colvin et al., 2019; Isaac et al., 2018). One South American mountaintop ecosystem, embedded within the Cerrado, Caatinga, and Atlantic Forest biomes, is the Campos Rupestres (CR, rocky grasslands). These areas are very old formations, with unfertile soils and communities adapted to them because of relatively stable ecological conditions over long geological time (Old Climatically‐Buffered Infertile Landscapes, OCBILs; Hopper, 2009). The CR occupies < 1% of Brazil and yet hosts 15% of its plant species, including 40% endemics (Silveira et al., 2019). The synergy among environmental filters, geographical barriers related to changes in altitude and slope, and interactions between species is an important driving force for a wide variety of floral and faunal adaptations and speciation in the CR (Castro et al., 2019; Fernandes, 2016; Fernandes et al., 2018; Silveira et al., 2016). The CRs have been destroyed at alarming rates and these disturbances are reflected in their springs and headwater streams (Callisto et al., 2019; Callisto, Solar, et al., 2019). The CRs are located in the oldest South American mountains and are characterized by endemism and relict populations. Their headwater streams and riparian zones form meta‐ecosystems forming natural corridors draining sky islands within the CR landscape matrix (Callisto, Solar, et al., 2019).

About 70%–80% of the total channel length in river basins is formed by low (1st–3rd) order streams (Wohl, 2017), and many of these originate on mountaintops (Callisto, Solar, et al., 2019), making them important for measuring beta biodiversity. Mountain headwater streams play a fundamental role in processing and transporting terrestrial and aquatic organic matter, and usually harbor high biodiversity (Boyero et al., 2016). Typically, aquatic communities in mountaintop headwater streams reflect adaptations to local environmental conditions (e.g., low temperature and nutrient availability, high current velocity or wind, high channel slopes, coarse substrates, physical habitat diversity, and riparian zone, and terrestrial ecosystem integrity) (Siebers et al., 2020). It is important to focus on both spatially extensive ecology (i.e., beta diversity) and site‐extent ecology (alpha diversity) because management of ecological systems must be extensive and local. But, there are simply too many possible sites to manage or study each one. Therefore, spatially extensive beta diversity studies have been valuable for determining patterns at national, continental, basin, biome, and ecoregion spatial extents in South American and North American countries (Dala‐Corte et al., 2020; Stoddard et al., 2008).

Most studies of diversity and ecological interactions in lotic ecosystems of tropical regions have traditionally been conducted in forest ecosystems; riverine systems crossing non‐forest ecosystems, including the CR, are seldom investigated (Linares et al., 2018). Thus, there is insufficient knowledge regarding how freshwater biodiversity is distributed in non‐forest montane streams. Given that these higher elevation streams connect upper to lower reaches in great river basins, such knowledge could support management strategies that would foster better conservation strategies for the headwaters of important South American river basins. Among the many taxa that compose the biodiversity in montane streams, benthic macroinvertebrates are good indicators of water quality, reflect altitudinal gradients (Castro et al., 2019) and participate in energy flux and nutrient cycling processes in freshwater ecosystems (Callisto, Solar, et al., 2019). To assess those macroinvertebrates, we focused our field sampling on leaf patches. Highly heterogeneous leaf patches and the biota living in them may promote turnover (species replacement) more than nestedness (specific subset of biota from the species pool) because each is driven to differing degrees by the regional species pool, species dispersal mechanisms, species interactions, landscape structure (elevation range, local relief and geomorphology), disturbance regimes, and interactions among these factors (Castro et al., 2020; Leal et al., 2018).

Therefore, the aim of this study was to assess the variation in taxonomic composition (measured as stream‐level beta diversity) among CR stream sites. Given that the turnover component made up most of the beta diversity in other Cerrado studies (e.g., Castro et al., 2020; Ligeiro et al., 2010; Pompeu et al., 2019) and that altitude and elevation range were the main drivers for structuring benthic macroinvertebrate assemblages (Gueuning et al., 2017; Musonge et al., 2020), we proposed two hypotheses. (a) Taxa replacement (i.e., turnover) should be the main reason for differences in benthic macroinvertebrate assemblages among stream sites; we predicted that turnover would be higher than nestedness in all streams. (b) Altitude and elevation range will be the main explanatory variables for differences in beta diversity, surpassing local site variables, which should account for only minor amounts of variation.

## 2 METHODS

### 2.1 Study area

**FIGURE 1:**
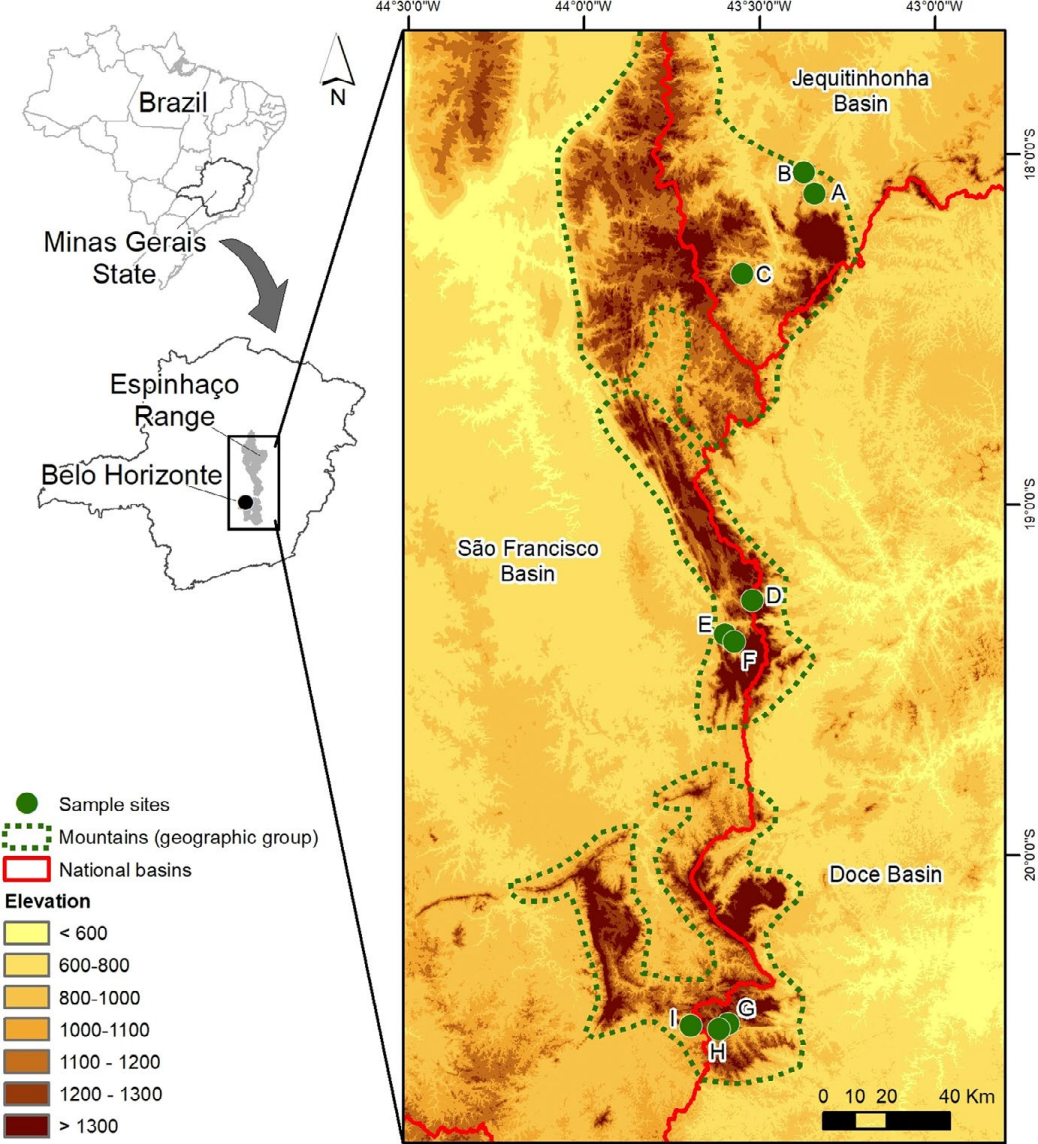
Map of the Espinhaço Meridional Mountain Range stream site locations (A–I) and their mountain areas (dotted lines). Sites A, B and C are in the Jequitinhonha River basin; sites E and F are in the São Francisco River basin and sites D, G, H and I are in the Doce River basin

The Espinhaço Meridional Mountain Range (EMMR) was recognized as a Biosphere Reserve by UNESCO in June 2005 (Conservation International, CI ‐ www.conservation.org.br) because of its importance to the natural and cultural heritage of Brazil and the world. The EMMR extends for ~400 km from south‐central Minas Gerais (Serra do Ouro Preto and Serra do Ouro Branco, SE) to north Minas Gerais, near the Diamantina municipality (Almeida‐Abreu, 1995), with widths of 50–100 km and altitudes of 700–1,800 m a.s.l. (Giulietti et al., 1987). The EMMR climate is tropical altitude (Cwb) with cool summers and a 5‐month dry season. Annual mean temperatures range from 17.5 to 18.5°C, and mean rainfall is between 1,450 and 1,600 mm/y (Alvares et al., 2013). The EMMR separates the São Francisco basin (to the west), the Doce basin (to the east), and the Jequitinhonha basin (to the northeast) through a mega‐geomorphologic cliff (Valadão, 2009). The EMMR is characterized by shallow and rocky soils, with frequent quartzite outcrops. Its geological history dates back to the end of the Paleoproterozoic (1.800 Ma), making it the second oldest mountain complex in Brazil (Almeida‐Abreu, 1995) and extremely old biogeographically (Castro et al., 2019). Vegetation types include unique rocky grasslands (CRs), Cerrado (Neotropical Savanna), and gallery forests that vary with elevation, longitude and latitude (Silveira et al., 2016). We selected nine CR stream sites distributed in a matrix of natural native vegetation and having over 70% canopy cover through use of an ad hoc selection based on access, level of conservation of riparian vegetation and minimum human disturbance. Three sites are in Rio Preto State Park, three are in Serra do Cipó National Park, and three are in Serra do Ouro Branco (Figure 1; Appendix S1, all appendices are available in Dryad: https://doi.org/10.5061/dryad.0p2ngf20g.).

### 2.2 Abiotic data collection

In each sampling season and in each stream site, we measured water pH and conductivity (µS/cm) with a portable multiparameter meter (YSI Multiprobe). Water samples were placed in an ice chest and taken to the laboratory where dissolved oxygen (mg/L), total nitrogen (mg/L), total phosphorus (µg/L), and turbidity (NTU) were determined via standard methods (APHA, 2005). We also measured instantaneous discharge using a current meter at a cross‐section having nonturbulent or near‐laminar flow in or near the studied sites (Peck et al., 2006).

Each stream was assigned to its Hydrographic Basin and Mountain (i.e., geographic group). We calculated altitude of the site (m), catchment area (km^2^), slope of the catchment (m; mean and range), and elevation of the catchment (m; mean and range) using GIS procedures and a SRTM model (~30 m spatial resolution; USGS, 2015) and determined each catchment's lithology (CODEMIG, 1997, 2017). We used data from WorldClim (Fick & Hijmans, 2017) to estimate catchment precipitation (mm; mean and range) and air temperature (°C; mean and range).

### 2.3 Biological data collection

In a 100 m long reach of each of the nine stream sites, two sampling stations were set where we collected as targeted samples, with a Surber sampler (30 × 30 cm; 250 μm mesh), five leaf patches in riffles, and five in pools in both dry (July 2006) and rainy (March 2007) seasons, totaling 180 samples. In pool habitats, we used a Surber sampler simply to ensure the same collection area in pools as riffles, placed leaf deposits into the net by hand and transferred the net contents into plastic bags for subsequent processing. Leaf patches were chosen because, as Ligeiro et al. (2020) reported, they concentrate high abundance and diversity of macroinvertebrates, acting as biodiversity hotbeds. Consequently, they limit assemblage responses to the disturbance gradient when this substrate is targeted, as opposed to using multi‐habitat sampling. At the sites, leaves were washed with stream water over a sieve (mesh size: 250 μm), separated manually and then the macroinvertebrates and fine particulate organic matter were placed in plastic jars and fixed with 70% ethanol. In the laboratory, invertebrates were identified to family using taxonomic keys (Costa et al., 2006; Fernández & Domínguez, 2001; Merritt & Cummins, 1996; Mugnai et al., 2010; Pérez, 1988).

### 2.4 Data analyses

We ran a permutational multivariate analysis of variance (PERMANOVA) pairwise contrasts analysis to test if benthic macroinvertebrate assemblages in pools and riffles in the rainy and dry seasons showed different taxonomic compositional profiles. Because we found insignificant variation between seasons and habitat types, we pooled all the samples at each site together. As complementary analyses we also ran NMDS using Bray–Curtis distance and PERMANOVA pairwise contrasts analyses to assess the assemblage composition variation among sampling sites (Anderson, 2017; Cáceres & Legendre, 2009; Wasserstein & Lazar, 2016). These analyses were run using the *vegan* package (Oksanen et al., 2017). We computed average pairwise beta diversity among sampling stations and seasons within each of the nine stream sites, with the Jaccard (presence‐absence) and Bray–Curtis (abundance) indices (Baselga, 2010, 2012), following the methodology described in Baselga and Orme (2012). We then calculated the percentage contribution of taxa replacement (i.e., turnover) and nestedness for all nine stream sites following Baselga and Orme (2012). All beta diversity analyses were calculated using the *betapart* package (Baselga & Orme, 2012) in R (R Development Core Team, 2017).

Because the stream site locations may have resulted in spatial autocorrelation, we ran Moran's I tests for spatial autocorrelation (Lecocq et al., 2019; Smeraldo et al., 2020), for both the Jaccard (family presence‐absence) and Bray–Curtis (family relative abundance) indices. Spatial autocorrelation was not significant among our stream sites for Jaccard (*p* = 0.69) and Bray–Curtis (*p* = 0.64) indices. For these analyses we used the *ape* package (Paradis & Schliep, 2019) in R.

To determine which were the main explanatory variables for beta diversity differences among stream sites, we used all the abiotic variables (Appendix S3) as predictor variables in two generalized linear models (GLMs) with a Gaussian distribution, where the Jaccard and Bray–Curtis values for each of the nine stream sites were the response variables. We then constructed a multimodel selection procedure based on all possible additive variable combinations that may have influenced beta diversity variation and calculated the cumulative AICc weights (*w*+) for each variable to determine which were the most likely variables (*w*+ ≥ 0.50) to have influenced beta diversity variation (Burnham & Anderson, 2002). Statistical analyses were implemented using the *MuMIn* package (Bartón, 2019) in R.

## 3 RESULTS

**FIGURE 2:**
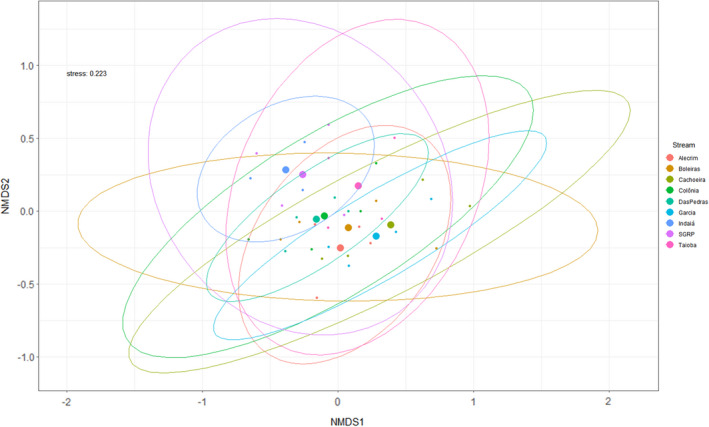
Non‐metric multidimensional scaling (NMDS) of Bray–Curtis similarity results for benthic macroinvertebrate assemblages of the Espinhaço Meridional Mountain Range sites. Stress level is 0.223. Ellipses circumscribe the site comparisons; large dots indicate the centroids of each ellipse of the same color

**FIGURE 3:**
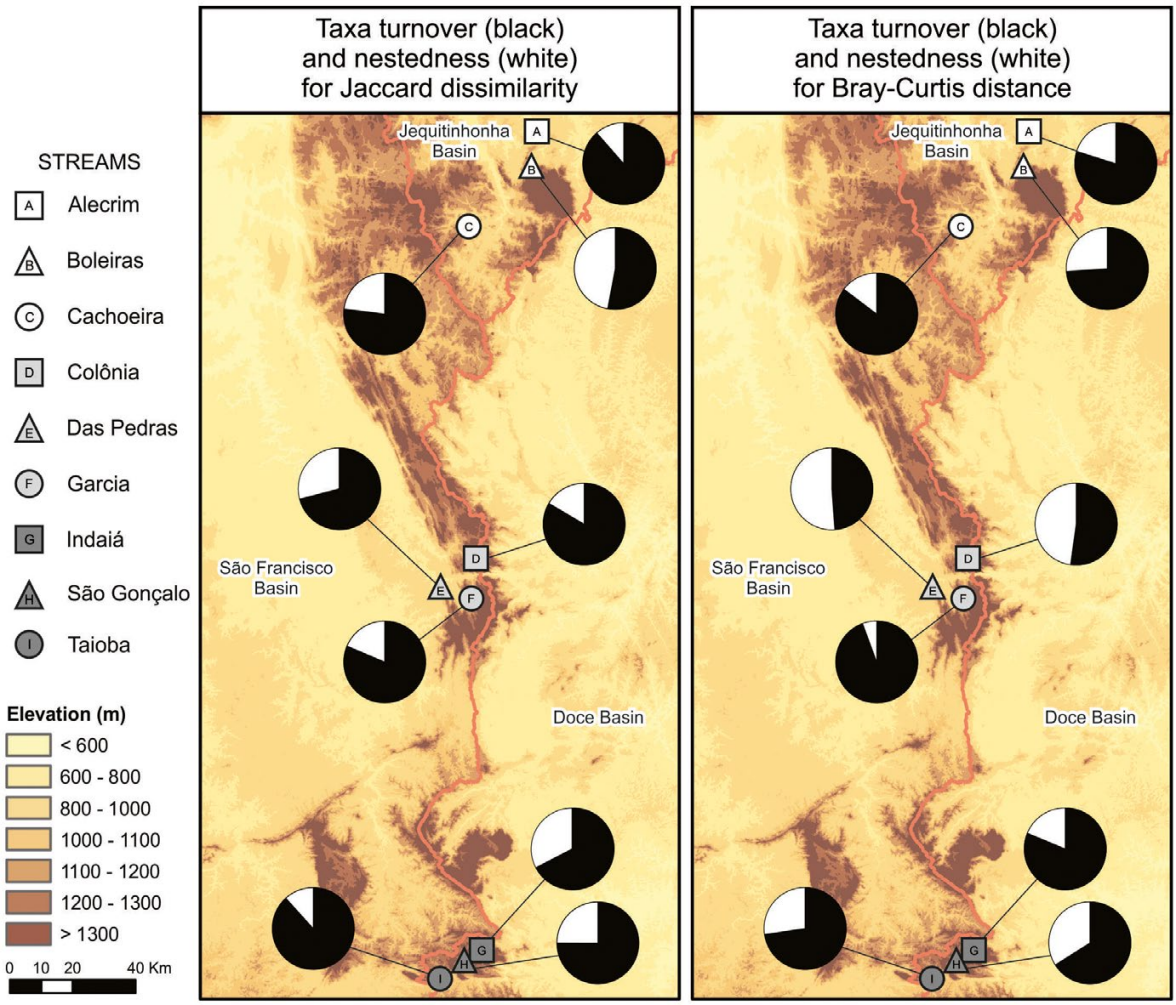
Proportion of taxa turnover and nestedness for Jaccard dissimilarity (left map) and Bray–Curtis distance (right map) for average pairwise beta diversity among sampling sites and seasons in each stream site

We found a total of 17,431 individuals and identified 63 taxa (Appendix S4). Chironominae, Simuliidae, Tanypodinae, Orthocladiinae (Diptera), and Elmidae (Coleoptera) were the most abundant families and subfamilies. Some sites were weakly to moderately similar to others (Figure 2, Appendix S2). The two sites that were most different from each other (Indaiá, Garcia) are in the same basin and at slightly different elevations. The pairs of sites that were most similar to each other (Colonia – Das Pedras, Indaiá – SGRP, Alecrim – Boleiras, Cachoeira – Garcia) are sometimes in the same basin and sometimes not in the same basin, but usually at similar elevations. The turnover component of beta diversity was higher for most stream sites (Figure 3; Appendix S5). For the Jaccard index, turnover was higher in all stream sites, varying from 52.97% to 88.57% of the dissimilarity. For the Bray–Curtis index, turnover was lower in only one of the nine stream sites (Das Pedras), reaching only 48.87%, whereas it was higher in all the other stream sites, varying between 52.20% and 94.38%.

For our second hypothesis, we found that the importance of environmental variables varied depending on the beta diversity index considered (Table 1). No variable showed significant influence on the variation in the Jaccard dissimilarity index, however, the Bray–Curtis distance index was negatively related to elevation range and positively related to dissolved oxygen.

**TABLE 1:** Cumulative AICc weights (*w*+) and estimates of variable coefficients (*β*) for predictor variables used to model the contributions to beta diversity (Jaccard dissimilarity and Bray–Curtis distance), of aquatic macroinvertebrates in nine Campos Rupestres (CR) stream sites in the Espinhaço Meridional Mountain Range (EMMR; Minas Gerais, Brazil)

Metric	Jaccard		Bray–Curtis	
	*w*_+_	*β*	*w*_+_	*β*
Altitude	0.11	—	<0.01	—
Discharge	0.37	—	<0.01	—
Dissolved Oxygen	0.03	—	**0.50**	**0.0619**
pH	0.12	—	0.47	—
Conductivity	0.02	—	<0.01	—
Turbidity	0.02	—	0.01	—
Total Nitrogen	0.09	—	<0.01	—
Total Phosphorus	0.02	—	<0.01	—
Hydrographic Basin	0.12	—	<0.01	—
Mountain	0.02	—	<0.01	—
Lithology	0.05	—	<0.01	—
Catchment Area	0.03	—	0.08	—
Slope mean	0.22	—	<0.01	—
Slope range	0.02	—	<0.01	—
Temperature mean	0.02	—	0.02	—
Temperature range	0.21	—	<0.01	—
Precipitation mean	0.08	—	<0.01	—
Precipitation range	0.06	—	<0.01	—
Elevation mean	0.02	—	0.39	—
Elevation range	0.15	—	**0.97**	**−0.0004**

Note

Values of w + in bold are those considered to be more likely (*w*+ ≥ 0.50) to have influenced beta diversity variation. Estimates of variable effects are based on the most parsimonious model that included that variable and are given only for variables with *w*+ ≥ 0.50.

## 4 DISCUSSION

Our first hypothesis, that taxa turnover would be the main reason for differences in macroinvertebrate assemblages among stream sites was confirmed because beta diversity was mostly due to turnover in all but one stream site for Bray–Curtis distance. The prevalence of turnover as the main driver of beta diversity likely reflects the general trend of macroinvertebrate taxonomic composition to be influenced by the large amount of naturally rare taxa that are replaced within and among stream sites (Castro et al., 2020; Perez‐Rocha et al., 2019). Rare taxa are mostly sensitive and specialist taxa, and therefore more likely to be affected by subtle differences in environmental conditions among stream sites (Arscott et al., 2006; Monaghan et al., 2005). Our results suggest that these Neotropical savanna montane stream sites may act as both reservoirs and refuges for rare or endemic macroinvertebrate taxa, highlighting their uniqueness for biodiversity conservation in a global change scenario.

Our second hypothesis, that catchment variables (i.e., altitude and elevation range) would be the main explanatory variables for diversity differences among stream sites, was partially corroborated. Similar to Castro et al. (2019), we found that elevation range was a more important driver for Bray–Curtis beta diversity among stream sites than dissolved oxygen. Other authors have reached different conclusions for fish in Amazonian streams (Leal et al., 2018), macroinvertebrates in Cerrado streams (Castro et al., 2018; Firmiano et al., 2021), and fish in Cerrado streams (Pompeu et al., 2019). Different results also have been reported for tropical streams in Malaysia (Al‐Shami et al., 2013), subtropical streams in South Africa (Rivers‐Moore, 2012) and for variability in MMI (multimetric index) scores of 3,420 temperate (USA) stream and river sites (Herlihy et al., 2020). Our results agree with those of Macedo et al. (2014), who reported that catchment‐extent predictors explained more of the variability in macroinvertebrate and fish taxa richness for lower elevation Cerrado sites. However, Leitão et al. (2018) found that local and catchment variables predicted similar amounts of fish species richness variability in Amazonian streams. Such differences likely arise for multiple reasons, including the spatial extent of the study area, the number of stream sites studied, the degrees of variability in the catchment‐extent and local‐extent predictor variables, interactions between catchment and local predictor variables, and the types of statistical analyses employed (Infante et al., 2019; Mostafavi et al., 2019; Wang et al., 2006).

The NMDS and Permanova analyses results reinforce the arguments of Moya et al. (2011), Stoddard et al. (2008) and Omernik et al. (2017) that ecoregional‐type drivers are stronger biotic drivers than basin drivers. Nonetheless, many regional aquatic ecologists favor river basins as critical drivers of biota (e.g., Abell et al., 2008), and they are to some degree. But at both larger and smaller spatial extents, as in our study, moderate differences in elevation range encompass other environmental variables that are associated with elevation range, such as catchment size, temperature, precipitation, DO, channel slope, and substrate size, which are often more important drivers of assemblage composition and richness than basin (Pont et al., 2009). Therefore, such drivers are frequently used for calibrating metrics employed in multimetric indices globally to increase their precision and assessment accuracy (Chen et al., 2019; Hering et al., 2006; Moya et al., 2011; Ruaro et al., 2020; Silva et al., 2017; Stoddard et al., 2008).

The lack of a significant result for the Jaccard dissimilarity index is likely because the studied stream sites are minimally disturbed reference streams and that presence/absence data are known to overvalue rare species (Jost, 2007), common in such streams (Martins et al., 2018). These ecosystems can show similar assemblage structure across different biomes (Santos et al., 2019). The natural variation found in reference streams may not be enough to affect patterns of family presence/absence. In contrast, abundances, evaluated through the Bray–Curtis distance index did detect variances, indicating that it is more sensitive to natural variability.

The leaves that fall from riparian vegetation are an important source of energy and nutrients for the metabolism of headwater streams. In the Neotropical Savanna (Cerrado biome), leaf fall is continuous throughout the year, with greater accumulation at the end of the dry season (Tonin et al., 2017). These leaves accumulate on the streambed, forming habitats that are important for aquatic macroinvertebrates, where they find food and shelter against predators (Mendes et al., 2017). Often, micro‐scale habitat features (e.g., plant species composition, age, chemical composition, leaf area, ash‐free dry mass of leaf patches per unit area) may influence macroinvertebrate assemblage composition (Graça, 2001), but they were not measured in this study. Also, Macedo et al. (2014) reported that landscape‐extent predictor variables accounted for more of the variation in macroinvertebrate taxa richness than did local site variables. Leaf patches represent temporary islets where the biodiversity of aquatic macroinvertebrates is high (biodiversity hotbeds), concentrating greater taxa richness, biomass, and abundance (Ligeiro et al., 2020).

Highly heterogeneous leaf patches and the biota living in them may promote turnover (species replacement) more than nestedness (specific subset of biota from the species pool), although both are driven by the regional species pool, species dispersal mechanisms, species interactions, landscape structure, disturbance regimes, and interactions among these factors (Castro et al., 2020; Leal et al., 2018). Leaf patches tend to be more heterogeneous in terms of plant species composition, age, chemical composition, leaf area, and edibility (Graça, 2001) compared with any single mineral substrate; therefore, the biotic heterogeneity in them favors greater species extirpation and replacement versus supporting a specific subset of the species pool. Likewise, macroinvertebrate dispersal mechanisms on montane streams do not favor any specific subset of the macroinvertebrate species pool to arrive at and then colonize a leaf patch that is in continuous flux, as opposed to a more stable mineral substrate such as gravel or cobble. Lastly, species interactions in a matrix of organic materials of varying ages, consistency and interstices sizes are likely to favor greater taxa turnover than any single mineral substrate (Ligeiro et al., 2020).

Weak fluvial connectivity among different CR streams helped ensure that aquatic macroinvertebrates that live in leaf patches in headwater streams showed high beta diversity. In fact, such areas in tropical montane streams have already been reported as sky islands for biodiversity (Gueuning et al., 2017). This is because, the same elevation across different sky islands supports more similar taxa than different elevations within the same stream. For example, Villamarin et al. (2021) reported that Chironomidae subfamilies and water quality also were associated with elevation bands instead of river basins in southwestern Ecuadorean Andean streams. The CR elevation gradients reflect environmental conditions that are steep and geologically stable, thereby supporting ecological and genetic differentiation, as proposed by von Humboldt over two centuries ago (Callisto, Solar, et al., 2019; Gueuning et al., 2017; Nicolson, 1987).

The waters of mountain streams combine to form hydrographic basins that connect landscapes along the river continuum, supplying ecosystem goods and services to half the human population on Earth (Callisto, Solar, et al., 2019). The beta diversity of aquatic macroinvertebrates associated with leaf patches in headwater streams of the Espinhaço Meridional Mountain Range represents important information in response to global warming and supports three conservation practices. (a) Maintaining riparian vegetation is an important legal obligation by national law (Federal Brazilian Law #12651/2012). (b) Conserving headwater streams favors the conservation of rare species in protected area networks that maintain biotic diversity and ecological processes (Socolar et al., 2015). (c) Guaranteeing water supplies for human populations in the São Francisco, Doce and Jequitinhonha River basins can directly assist conservation planning in Neotropical mountain streams.

## CONFLICT OF INTEREST

The authors have no conflicts of interest to declare.

## AUTHOR CONTRIBUTIONS

**Marcos Callisto:** Conceptualization (equal); data curation (equal); funding acquisition (equal); project administration (equal); supervision (equal); validation (equal); writing – original draft (equal). **Marden S. Linares:** Conceptualization (equal); formal analysis (equal); investigation (equal); validation (equal); writing – review and editing (equal). **Walace P. Kiffer:** Conceptualization (equal); formal analysis (supporting); investigation (equal); writing – review and editing (equal). **Robert M. Hughes:** Conceptualization (equal); investigation (supporting); writing – review and editing (equal). **Marcelo S. Moretti:** Conceptualization (equal); investigation (equal); writing – review and editing (equal). **Diego R. Macedo:** Conceptualization (equal); investigation (equal); writing – review and editing (equal). **Ricardo Solar:** Conceptualization (equal); formal analysis (equal); investigation (equal); validation (equal); writing – review and editing (equal).

## Supporting information

Appendix S1‐S2

Appendix S3

Appendix S4

Appendix S5

## Data Availability

All data used in the production of this article are available via Dryad: https://doi.org/10.5061/dryad.0p2ngf20g.
