# Supplementary material for: Beta diversity of aquatic macroinvertebrate assemblages associated with leaf patches in neotropical montane streams
Source: Ecol Evol. 2021 Feb 7;11(6):2551–60. doi: 10.1002/ece3.7215 (PMC8009175; doi:10.1002/ece3.7215)
Supplement: Supplementary file 1 — Appendix S1‐S2 [file ECE3-11-2551-s002.docx]

Appendix 1. Geographical coordinates, local names, map codes (Figure 1), elevation and basins of the study sites.

| Coordinates | Stream | Map Code | Elevation (m) | Hydrographic Basins |
| --- | --- | --- | --- | --- |
|  |  |  |  |  |
| 23K 0675117 UTM 7996444 | Boleiras | A | 800 | Jequitinhonha River |
|  |  |  |  |  |
| 23K 0672097 UTM 8003175 | Alecrim | B | 740 | Jequitinhonha River |
|  |  |  |  |  |
| 23K 0653301 UTM 7971287 | São Gonçalo | C | 1120 | Jequitinhonha River |
|  |  |  |  |  |
| 23K 0647010 UTM 7857537 | Das Pedras | E | 760 | São Francisco River |
|  |  |  |  |  |
| 23K 0649831 UTM 7855164 | Taioba | F | 750 | São Francisco River |
|  |  |  |  |  |
| 23K 0655422 UTM 7868224 | Indaiá | D | 1200 | Doce River |
|  |  |  |  |  |
| 23K 0647125 UTM 7734564 | Cachoeira | G | 940 | Doce River |
|  |  |  |  |  |
| 23K 0644326 UTM 7732899 | Garcia | H | 980 | Doce River |
|  |  |  |  |  |
| 23K 0635853 UTM 7733991 | Colônia | I | 1320 | Doce River |
|  |  |  |  |  |

Appendix 2: PERMANOVA pairwise contrasts analysis. Values in bold indicate significant composition differences among sites at p < 0.05 (bold, explaining 29-43% of the variability), p < 0.10 (italic, explaining 23-28% of the variability), p < 0.20 (underlined, explaining 20-29% of the variability).

| pairs | *F* | R^2^ | *p* |
| --- | --- | --- | --- |
| Alecrim vs Boleiras | 1.8182 | 0.23 | *0.06* |
| Alecrim vs SGRP | 2.125333 | 0.26 | *0.09* |
| Alecrim vs Indaiá | 4.447525 | 0.43 | **0.03** |
| Alecrim vs DasPedras | 2.105084 | 0.26 | 0.11 |
| Alecrim vs Taioba | 3.558466 | 0.37 | **0.03** |
| Alecrim vs Cachoeira | 2.195618 | 0.27 | 0.12 |
| Alecrim vs Garcia | 1.610228 | 0.21 | 0.17 |
| Alecrim vs Colônia | 1.604388 | 0.21 | 0.15 |
| Boleiras vs SGRP | 1.011982 | 0.14 | 0.40 |
| Boleiras vs Indaiá | 2.124768 | 0.26 | *0.06* |
| Boleiras vs DasPedras | 0.628389 | 0.094 | 0.69 |
| Boleiras vs Taioba | 1.304849 | 0.18 | 0.28 |
| Boleiras vs Cachoeira | 0.719951 | 0.11 | 0.60 |
| Boleiras vs Garcia | 0.938835 | 0.14 | 0.42 |
| Boleiras vs Colônia | 0.936633 | 0.14 | 0.52 |
| SGRP vs Indaiá | 1.499949 | 0.20 | 0.17 |
| SGRP vs DasPedras | 0.800653 | 0.12 | 0.54 |
| SGRP vs Taioba | 0.846175 | 0.12 | 0.58 |
| SGRP vs Cachoeira | 0.931423 | 0.13 | 0.37 |
| SGRP vs Garcia | 2.047158 | 0.25 | 0.14 |
| SGRP vs Colônia | 1.009488 | 0.14 | 0.43 |
| Indaiá vs DasPedras | 2.014881 | 0.25 | 0.12 |
| Indaiá vs Taioba | 2.396073 | 0.29 | **0.03** |
| Indaiá vs Cachoeira | 2.282522 | 0.28 | *0.06* |
| Indaiá vs Garcia | 3.916768 | 0.40 | **0.03** |
| Indaiá vs Colônia | 3.158758 | 0.35 | **0.03** |
| DasPedras vs Taioba | 1.081149 | 0.15 | 0.49 |
| DasPedras vs Cachoeira | 0.968517 | 0.14 | 0.41 |
| DasPedras vs Garcia | 1.64761 | 0.22 | 0.15 |
| DasPedras vs Colônia | 1.041742 | 0.15 | 0.43 |
| Taioba vs Cachoeira | 0.858774 | 0.13 | 0.45 |
| Taioba vs Garcia | 2.465851 | 0.29 | 0.11 |
| Taioba vs Colônia | 1.728495 | 0.22 | 0.17 |
| Cachoeira vs Garcia | 1.542448 | 0.20 | 0.20 |
| Cachoeira vs Colônia | 1.373825 | 0.19 | 0.29 |
| Garcia vs Colônia | 1.234647 | 0.17 | 0.26 |
